# Breath-synchronized electrical stimulation of the expiratory muscles in mechanically ventilated patients: a randomized controlled feasibility study and pooled analysis

**DOI:** 10.1186/s13054-020-03352-0

**Published:** 2020-10-30

**Authors:** Annemijn H. Jonkman, Tim Frenzel, Euan J. McCaughey, Angus J. McLachlan, Claire L. Boswell-Ruys, David W. Collins, Simon C. Gandevia, Armand R. J. Girbes, Oscar Hoiting, Matthijs Kox, Eline Oppersma, Marco Peters, Peter Pickkers, Lisanne H. Roesthuis, Jeroen Schouten, Zhong-Hua Shi, Peter H. Veltink, Heder J. de Vries, Cyndi Shannon Weickert, Carsten Wiedenbach, Yingrui Zhang, Pieter R. Tuinman, Angélique M. E. de Man, Jane E. Butler, Leo M. A. Heunks

**Affiliations:** 1 Department of Intensive Care Medicine, Amsterdam University Medical Centers, location VUmc, Postbox 7505, 1007 MB Amsterdam, The Netherlands; 2 Amsterdam Cardiovascular Sciences Research Institute, Amsterdam UMC, Amsterdam, The Netherlands; 3 Department of Intensive Care Medicine, Radboud University Medical Center, Nijmegen, The Netherlands; 4 Neuroscience Research Australia, 139 Barker Street, Randwick, NSW 2031 Australia; 5 School of Medical Sciences, University of New South Wales, Kensington, NSW 2052 Australia; 6 Liberate Medical LLC, Crestwood, KY 40014 USA; 7 Prince of Wales Hospital, Randwick, NSW 2031 Australia; 8 Department of Intensive Care Medicine, Canisius Wilhelmina Hospital, Nijmegen, The Netherlands; 9 Cardiovascular and Respiratory Physiology Group, Technical Medical Centre, University of Twente, Enschede, The Netherlands; 10 Department of Biomedical Signals and Systems, Technical Medical Centre, University of Twente, Enschede, The Netherlands; 11 School of Psychiatry, University of New South Wales, Kensington, NSW 2052 Australia; 12 Department of Neuroscience and Physiology, Upstate Medical University, New York, 13210 USA

**Keywords:** Functional electrical stimulation, Expiratory muscles, Mechanical ventilation

## Abstract

**Background:**

Expiratory muscle weakness leads to difficult ventilator weaning. Maintaining their activity with functional electrical stimulation (FES) may improve outcome. We studied feasibility of breath-synchronized expiratory population muscle FES in a mixed ICU population (“Holland study”) and pooled data with our previous work (“Australian study”) to estimate potential clinical effects in a larger group.

**Methods:**

*Holland:* Patients with a contractile response to FES received active or sham expiratory muscle FES (30 min, twice daily, 5 days/week until weaned). Main endpoints were feasibility (e.g., patient recruitment, treatment compliance, stimulation intensity) and safety. *Pooled:* Data on respiratory muscle thickness and ventilation duration from the Holland and Australian studies were combined (*N* = 40) in order to estimate potential effect size. Plasma cytokines (day 0, 3) were analyzed to study the effects of FES on systemic inflammation.

**Results:**

*Holland:* A total of 272 sessions were performed (active/sham: 169/103) in 20 patients (*N* = active/sham: 10/10) with a total treatment compliance rate of 91.1%. No FES-related serious adverse events were reported. *Pooled:* On day 3, there was a between-group difference (*N* = active/sham: 7/12) in total abdominal expiratory muscle thickness favoring the active group [treatment difference (95% confidence interval); 2.25 (0.34, 4.16) mm, *P* = 0.02] but not on day 5. Plasma cytokine levels indicated that early FES did not induce systemic inflammation. Using a survival analysis approach for the total study population, median ventilation duration and ICU length of stay were 10 versus 52 (*P* = 0.07), and 12 versus 54 (*P* = 0.03) days for the active versus sham group. Median ventilation duration of patients that were successfully extubated was 8.5 [5.6–12.2] versus 10.5 [5.3–25.6] days (*P* = 0.60) for the active (*N* = 16) versus sham (*N* = 10) group, and median ICU length of stay was 10.5 [8.0–14.5] versus 14.0 [9.0–19.5] days (*P* = 0.36) for those active (*N* = 16) versus sham (*N* = 8) patients that were extubated and discharged alive from the ICU. During ICU stay, 3/20 patients died in the active group versus 8/20 in the sham group (*P* = 0.16).

**Conclusion:**

Expiratory muscle FES is feasible in selected ICU patients and might be a promising technique within a respiratory muscle-protective ventilation strategy. The next step is to study the effects on weaning and ventilator liberation outcome.

*Trial registration:* ClinicalTrials.gov, ID NCT03453944. Registered 05 March 2018—Retrospectively registered, https://clinicaltrials.gov/ct2/show/NCT03453944.

## Background

The respiratory muscle pump drives alveolar ventilation. The diaphragm is the most prominent inspiratory muscle, while abdominal wall muscles play an important role in active expiration [1, 2]. Respiratory muscle weakness is highly prevalent in critically ill intensive care unit (ICU) patients and associated with difficult ventilator weaning, prolonged ICU stay, and mortality [3–10]. Mechanical ventilation is a critical contributor as excessive ventilator assist may result in diaphragm disuse atrophy [3, 4, 11]. Indeed, maintaining diaphragm activity during mechanical ventilation may preserve diaphragm function [4]. Moreover, in a preclinical study, electrical stimulation of the phrenic nerves was shown to attenuate diaphragm atrophy resulting from controlled mechanical ventilation [12].

In contrast, the impact of mechanical ventilation on the expiratory muscles is less well studied and interventions targeting these muscles are lacking. This is surprising, as the expiratory muscles are vital for airway clearance, prevention of atelectasis, and enhancement of minute ventilation in conditions of low inspiratory muscle capacity and/or high respiratory load [1]. Development of expiratory muscle weakness during ICU stay has been associated with extubation failure and (re)hospitalization due to respiratory complications [9, 10, 13, 14]. Maintaining their activity under mechanical ventilation is therefore likely to improve outcome.

Functional electrical stimulation (FES) of limb muscles has been studied extensively as a strategy to limit disuse atrophy and to improve muscle function during prolonged immobilization [15, 16]. FES uses electrical currents to generate artificial skeletal muscle contraction without patient cooperation, making it an attractive intervention in uncooperative mechanically ventilated patients. Recently, we published results of a pilot study employing noninvasive breath-synchronized FES of the expiratory abdominal wall muscles, further referred to as ‘expiratory muscle FES’, in 20 ICU patients (“Australian study”) [17] and showed that this intervention has potential benefits. However, the active group of this single-center study consisted mainly of neurocritically ill patients (70%) and results need to be confirmed in a more diverse ICU population. The aim of the current study (“Holland study”) was to assess feasibility of performing expiratory muscle FES in a mixed ICU population. Additionally, data from both studies were pooled to estimate potential clinical effects in a larger, more heterogeneous patient group.

## Methods

See Additional file 1 for additional details.

### Holland study

#### Participants and Study design

Twenty patients were randomized in this prospective sham-controlled feasibility study conducted in three mixed ICUs in the Netherlands (Radboud University Medical Center; Amsterdam UMC, location VUmc; Canisius Wilhelmina Ziekenhuis). Patients were enrolled as early as possible but within 72 h after intubation. Exclusion criteria were an anticipated stay on the ventilator < 72 h at the time of study enrolment, congenital myopathies or neuropathies, and contraindications for expiratory muscle FES (cardiac pacemaker, refractory epilepsy, recent (< 4 weeks) abdominal surgery, body mass index > 35 kg/m^2^, and pregnancy). Written informed consent was obtained from the patient’s substitute decision-maker. Only patients with an adequate contractile response to expiratory muscle FES using stimulation settings similar as for the active group (see below and Additional file 1) were randomized.

#### Expiratory muscle FES

Expiratory muscle FES was applied for 30 min, twice daily, for 5 days per week (first 5 days consecutively), until patients were weaned from mechanical ventilation, but no longer than six weeks. Local protocols for mechanical ventilation and weaning were followed.

Stimulation was applied during exhalation using an investigational device (VentFree model VK03-K, Liberate Medical LLC, USA) (Additional file 1: Figure 1 and [17]) via surface electrodes on the abdominal wall, and stimulation intensity was titrated in order to activate the external oblique, internal oblique and transversus abdominis muscles (Additional file 1: Figure 2). The patient’s tolerance of expiratory muscle FES was continuously monitored by means of clinical judgment; sessions were discontinued in the presence of stop criteria (see Additional file 1). In the active group (*N* = 10), settings were as follows: frequency 30 Hz, pulse width 352 µs and a current amplitude (intensity) set to cause strong muscle contraction, with a maximum intensity initially set at 60 mA (tolerated intensity in healthy volunteers [18]). After the first four patients were enrolled in the study, the protocol was amended to allow a maximum intensity of 100 mA (maximum device output). Strong muscle contraction was verified (visible and palpable) every ten minutes throughout each FES session, and if necessary, stimulation intensity was increased. In the sham group (*N* = 10), the following settings were applied and would allow a patient to have a sensation of stimulation without muscle contraction (as verified clinically and with ultrasound): frequency 10 Hz, pulse width 352 µs, intensity 10 mA.

#### Data collection

During each stimulation session, ventilator parameters and vital signs were collected. Ultrasound measurements were performed (see Additional file 1 and [19]) to study changes in respiratory muscle thickness (external oblique, internal oblique and transversus abdominis (their combined thickness is further referred to as *total abdominal expiratory muscle thickness*), rectus abdominis thickness and diaphragm thickness). The researcher administering expiratory muscle FES did not perform ultrasound recordings, and outcome assessors were not in the patient room when stimulation was delivered. Blood samples for the analysis of plasma cytokine levels were collected at baseline and on day 3 (see Additional file 1). Ventilation duration was collected as the number of days from mechanical ventilation onset until the first successful extubation [i.e., no need for ventilator support (no noninvasive ventilation and no high-flow nasal therapy) for 48 h after extubation].

#### Outcomes

The primary outcome was the feasibility of performing expiratory muscle FES in terms of patient recruitment, contractile response, and treatment compliance, and safety. As an additional safety endpoint, plasma cytokine levels were studied to evaluate whether early application of FES was associated with enhanced systemic pro-inflammatory response. This was evaluated for the full analysis set (per-protocol analysis). Ultrasound and clinical data of the Holland study were evaluated in a pooled analysis.

### Pooled analysis

Data from the Holland and Australian studies could be combined (*N* = 40) as FES protocols were similar until extubation. Pooled endpoints were respiratory muscle thickness, plasma cytokine levels, ventilation duration, ICU length of stay, and ICU mortality. Ultrasound data were combined for patients with a treatment compliance of ≥ 75% treatment days from the intent-to-treat set and until day 5 after enrollment, as ultrasound protocols were similar until this point. Other pooled endpoints were analyzed for the full analysis set (per-protocol analysis).

### Statistics

Data are presented as medians with interquartile range [*q*_1_–*q*_3_], mean ± standard deviation, or percentages. Statistical analyses were performed using two-sided hypothesis tests at the 5% significance level.

#### Holland study

As this is a feasibility study and no data were available on the effects of expiratory muscle FES on expiratory muscle thickness or function when designing the study, we planned to enroll a convenience sample of ten subjects per group, 20 in total. Compliance to expiratory muscle FES sessions was calculated as the percentage of all sessions that should have been completed between randomization and study completion or withdrawal and was considered a continuous variable. Stimulation intensities and safety endpoints (adverse events) are presented using descriptive statistics. Changes in plasma cytokine levels were assessed using a two-way ANOVA with factors of group (active, sham) and time (measurements at baseline and on day 3). Further details are presented in Additional file 1.

#### Pooled analysis

Survival analysis was performed to estimate the median ventilation duration and ICU length of stay, applying the cumulative incidence competing risks method. Competing risks were death or withdrawal of treatment (e.g., ventilator support) with the intention of subsequent death. Participants who were alive but who did not experience the event of interest were right censored at the last available study day. Gray’s test was used for comparison of cumulative incidence functions, and the median duration was defined as the time point where 50% of participants experienced the event of interest. In addition, we calculated the median [*q*_1_–*q*_3_] ventilation duration and ICU length of stay for those patients that experienced the event of interest during the study period. Differences between these groups were assessed with the Mann–Whitney *U* test, according to the distribution. Changes from baseline of total abdominal expiratory muscle thickness were analyzed with a linear mixed effects model with fixed factors of baseline thickness, group, assessment session and group by assessment session interaction, and a random effect of participant. For further statistical details and analyses of other outcome parameters, see Additional file 1.

## Results

### Holland study

#### Study population

**Fig. 1:**
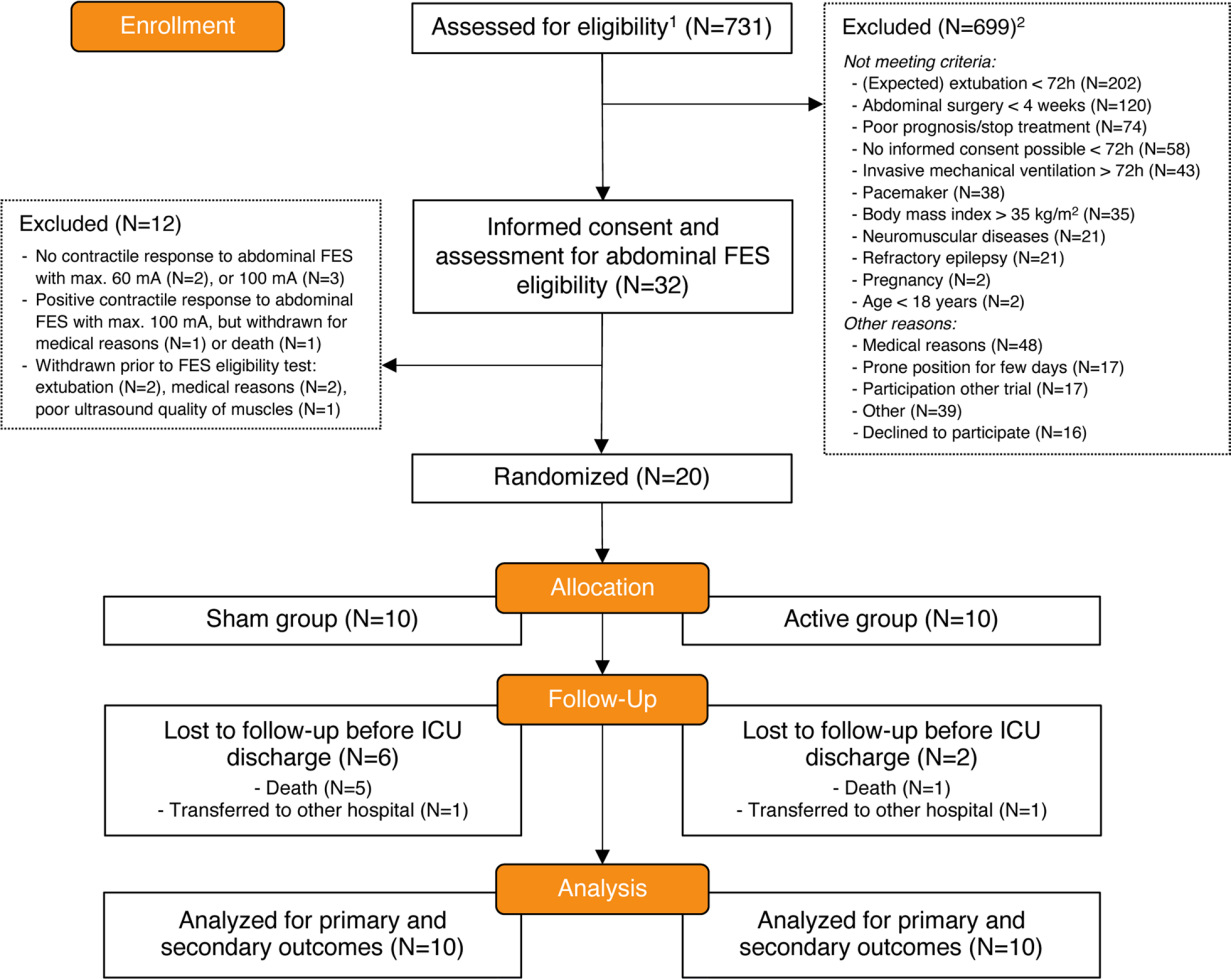
CONSORT flow diagram. Study flow diagram of patients admitted to the intensive care unit (ICU) and the randomization process between January 2017 and January 2019. ^1^Screening of all admitted ICU patients under invasive mechanical ventilation, on days when the study team was available. ^2^Patients could have fulfilled more than one exclusion criterion

Figure 1 presents the CONSORT flow diagram. Five patients did not show a contractile response to expiratory muscle FES (*N* = 2 at 60 mA and *N* = 3 at 100 mA) and were withdrawn before randomization. No potential explanatory factors hampering contractile response could be identified in these patients (i.e., no recent neuromuscular blockers, no relevant medical history, and body mass index, sedation, and fluid status were not different from other patients). Baseline characteristics of the randomized patients (*N* = active/sham: 10/10) are presented in Table 1.

**Table 1:** Patient characteristics for the Holland study and the pooled data (including the Holland and Australian study)

	Holland study			Pooled data (Holland + Australia)		
	All (*N* = 20)	Active (*N* = 10)	Sham (*N* = 10)	All (*N* = 40)	Active (*N* = 20)	Sham (*N* = 20)
Age, years	69.0 ± 8.5	72.2 ± 6.1	65.7 ± 9.6	63.4 ± 14	64.5 ± 14.9	62.4 ± 13.3
Sex, male/female	14/6	8/2	6/4	26/14	15/5	11/9
Body mass index, kg/m^2^	26.4 ± 4.4	25.9 ± 5.1	26.8 ± 3.8	26.4 ± 4.4	26.8 ± 4.5	26.5 ± 3.2
History of COPD, % (*n*/*N*)	50 (10/20)	60 (6/10)	40 (4/10)	25 (10/40)	30 (6/20)	20 (4/20)
MV duration at enrollment, days	2.5 ± 1.0	2.4 ± 1.0	2.5 ± 1.0	2.5 ± 0.8	2.5 ± 0.9	2.5 ± 0.8
FiO_2_ at study enrollment	0.42 ± 0.12	0.42 ± 0.09	0.42 ± 0.15	0.36 ± 0.13	0.34 ± 0.11	0.37 ± 0.15
Primary reason for MV, % (*n*/*N*)						
Cardiac arrest	5 (1/20)	0 (0/0)	10 (1/10)	2.5 (1/40)	0 (0/20)	5 (1/20)
Pneumonia	50 (10/20)	40 (4/10)	60 (6/10)	30 (12/40)	25 (5/20)	35 (7/20)
Postoperative	5 (1/20)	10 (1/10)	0 (0/0)	5 (2/40)	10 (2/20)	0 (0/20)
Exacerbation COPD	25 (5/20)	30 (3/10)	20 (2/10)	12.5 (5/40)	15 (3/20)	10 (2/20)
Neurologic dysfunction	10 (2/20)	10 (1/10)	10 (1/10)	32.5 (13/40)	40 (8/20)	25 (5/20)
Sepsis	0 (0/0)	0 (0/0)	0 (0/0)	12.5 (5/40)	5 (1/20)	20 (4/20)
Other	5 (1/20)	10 (1/10)	0 (0/0)	2.5 (2/40)	5 (1/20)	5 (1/20)
Successfully extubated during study, % (*n*/*N*)	60 (12/20)	80 (8/10)	40 (4/10)	65 (26/40)	80 (16/20)	50 (10/20)

Values are presented as mean ± standard deviation, or as absolute or percentage number of patients

*COPD* chronic obstructive pulmonary disease, *FiO*_*2*_ fraction of inspired oxygen, *ICU* intensive care unit, *MV* mechanical ventilation

#### Feasibility

Table 2 presents expiratory muscle FES session compliance and adverse events. Nonserious adverse events categorized as ‘possibly’ or ‘definitely’ related to the intervention were reported in sixteen sessions (*n* = active/sham sessions: 13/3, percentage of total sessions per group active/sham: 7.7%/2.9%) in a total of five active patients and in one sham patient. In the active group, these events included discomfort (*n* = 3 sessions, in two patients) and brief, spontaneous reversible episodes of hypertension and/or tachycardia (*n* = 10 sessions, in four patients) and were reasons for an early stop of the current expiratory muscle FES session (Table 2). Device-related adverse events reported in the sham group included hypertension and tachycardia (*n* = 3, in one patient). No FES-related serious adverse events were reported, as judged by a physician and confirmed by the local ethics board.

**Table 2:** Compliance to expiratory muscle functional electrical stimulation (FES) sessions and reported adverse events (AE) during the study period

Treatment compliance	Sham (*N* = 10)	Active (*N* = 10)	Total (*N* = 20)
Total sessions, *n*	103	169	272
30-min sessions completed, *n* (% of total sessions)	98 (95.1)	147 (87)	245 (91.1)
Reasons for early stop of current session^a^, % (*n*/*N*)			
Heart rate < 40 bpm	0 (0/103)	0 (0/169)	0 (0/272)
Heart rate > 130 bpm	1 (1/103)	0.6 (1/169)	0.7 (2/272)
MAP < 60 mmHg	0 (0/103)	0 (0/169)	0 (0/272)
MAP > 110 mmHg	1.9 (2/103)	6.5* (11/169)	4.8 (13/272)
Respiratory rate > 40 breaths/min	1 (1/103)	3 (5/169)	2.2 (6/272)
SpO_2_ < 90%	1 (1/103)	0 (0/169)	0.4 (1/272)
Behavior pain scale > 4	0 (0/103)	0.6 (1/169)	0.4 (1/272)
Patient request to stop	0 (0/103)	2.4 (4/169)	1.5 (4/272)
Treatment days from intent-to-treat set, % (median (*q*_1_–*q*_3_))	100 (69–100)	89 (50–100)	100 (50–100)
Reasons for treatment stop before extubation, patients % (*n*/*N*)			
Discomfort	10 (1/10)	10 (1/10)	10 (2/20)
Switch to NAVA mode^b^	10 (1/10)	20 (2/10)	15 (3/20)
Patient request to stop	0 (0/10)	10 (1/10)	5 (1/20)

Safety^c^	Event	Patient % (*n*/*N*)	Event	Patient % (*n*/*N*)	Event	Patient % (*n*/*N*)
*Adverse events (any)*	11	80 (8/10)	17	60 (6/10)	28	70 (14/20)
Unanticipated adverse device effects	0	0 (0/10)	9	40 (4/10)	9	20 (4/20)
AEs leading to discontinuation of current FES session^d^ or study	8	60 (6/10)	14	50 (5/10)	22	55 (11/20)
Serious AEs—total	7	60 (6/10)	3	20 (2/10)	10	40 (8/20)
Serious AEs—deaths	5	50 (5/10)	1	10 (1/10)	6	30 (6/20)
*Intervention-related adverse events*^*e*^	3	10 (1/10)	13	50 (5/10)	16	30 (6/20)
Unanticipated adverse device effects	0	0 (0/10)	9	40 (4/10)	9	20 (4/20)
AEs leading to discontinuation of current FES session^d^ or study	3	10 (1/10)	11	50 (5/10)	14	30 (6/20)
Serious AEs—total	0	0 (0/10)	0	0 (0/10)	0	0 (0/20)
Serious AEs—deaths	0	0 (0/10)	0	0 (0/10)	0	0 (0/20)

*MAP* mean arterial pressure, *SpO*_2_ oxygen saturation, *NAVA* neurally adjusted ventilatory assist

^*^Six of these episodes developed in one patient who already had a high MAP prior to the FES session

^a^Also counted as adverse event, see safety parameters

^b^Stimulation artifacts were visible in the diaphragm electrical activity (EAdi) signal, limiting the application of NAVA mode. As the study protocol did not allow to change ventilator mode, expiratory muscle FES sessions were discontinued in patients ventilated in NAVA.

^c^Participants that experienced multiple adverse events within a specific category are counted only once in the calculation of occurrence rate for that category

^d^Discontinuation of current expiratory muscle FES session based on stop criteria.

^e^Intervention-related adverse events include definitely, probably and possibly relationships

For the total population, the threshold intensity for strong muscle contraction was 70 [54–73] mA and the maximum tolerated intensity was 100 [60–100] mA, as assessed prior to randomization. Muscle contraction was confirmed during all active sessions. The average stimulation intensity remained stable over the study period (change of − 0.9 [− 2.1–0.0] %). The patient’s average session-to-session change in stimulation intensity (either an increase or decrease as compared to the previous session) was 4.8 [3.0–7.1] %. Additional file 1: Figure 3 shows that with higher Richmond Agitation-Sedation Scale, the applied stimulation intensity was lower compared to the patient’s average intensity (*P* = 0.02). Most active sessions were performed during pressure support ventilation (PSV) (percentage of sessions during PSV: 96.4 [59.6–100] %).

#### Inflammatory markers

**Fig. 2:**
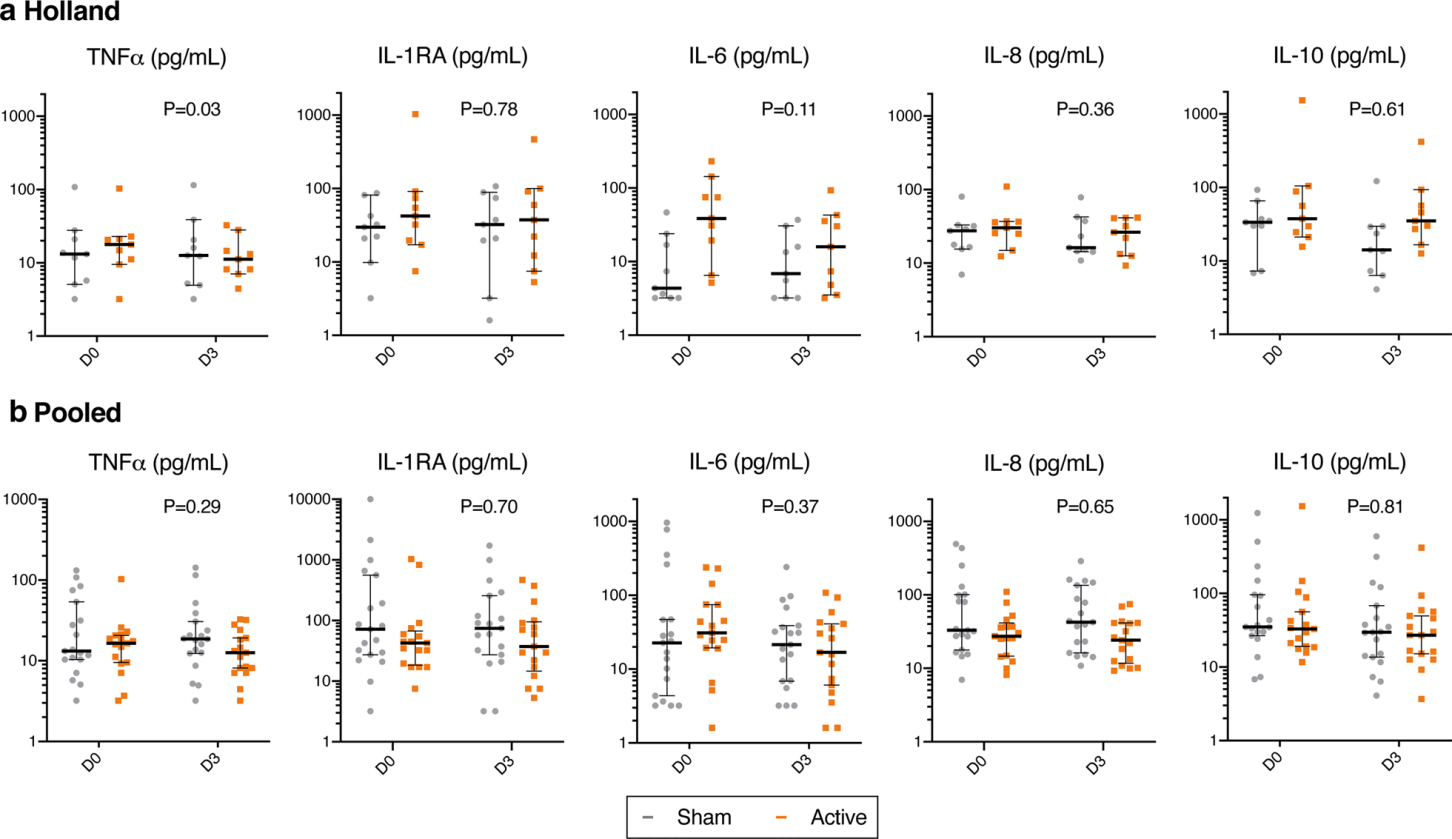
Systemic inflammatory markers obtained at study enrollment (baseline, D0) and on the third treatment day (D3). **a** Holland study (*N* = active/sham; 9/9), **b** pooled analysis (*N* = 17/19). Measured cytokines included tumor necrosis factor (TNF)-α, interleukin (IL)-1 receptor antagonist (RA), IL-6, IL-8, and IL-10. Levels of IL-1β were for 96% of the samples below the limit of quantitation and are therefore not presented. Data are presented as medians with interquartile ranges. Due to the large between-subject variability in inflammatory markers, data are presented on a logarithmic scale. Log-transformed data were analyzed using a two-way analysis of variance with factors of time (D0, D3) and group. *P* values represent the interaction effect of group × time

Figure 2a presents the plasma cytokine levels at baseline and on day 3 (*N* = active/sham; 9/9). There was a between-group difference in the change in pro-inflammatory marker TNF-α (*P* = 0.03), with a trend toward a decrease in TNF-α levels for the active group versus no change in the sham group. Mean differences compared to baseline were − 0.15 ± 0.20 pg/mL (*P* = 0.05) versus 0.02 ± 0.06 pg/mL (*P* = 0.32), respectively.

### Pooled analysis

See Table 1 for characteristics of the pooled study population (*N* = active/sham: 20/20).

#### Ultrasound

**Fig. 3:**
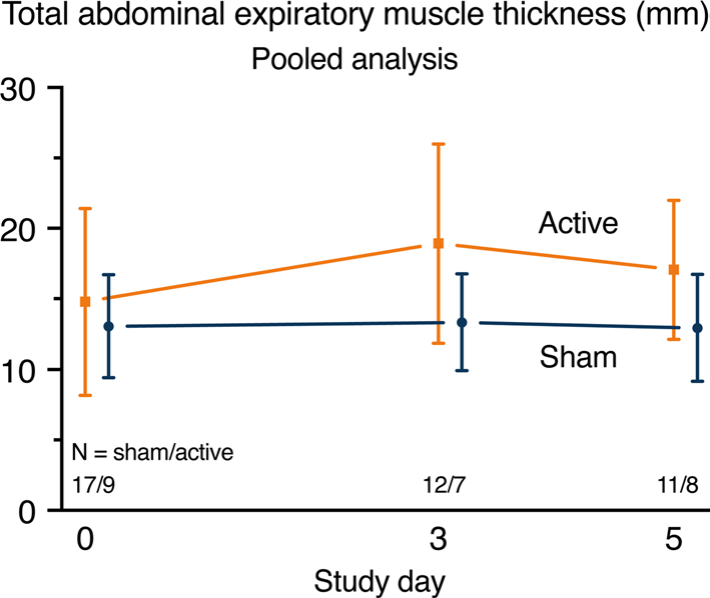
Pooled results for total abdominal expiratory muscle thickness changes over the first 5 days after randomization. On day 3, changes from baseline were different between groups as per a linear mixed model analysis (*P* = 0.02). Data represent the absolute means ± standard deviation

Due to technical issues, ultrasound data of four Holland study patients from one site were incomplete (two active and two sham patients). Missing data for the Australian study are addressed in [17]. In patients with adequate baseline measurements available and who completed ≥ 75% of stimulation sessions from the intend-to-treat set, total abdominal expiratory muscle thickness at baseline was 11.7 [10.6–20.6] versus 12.4 [10.5–15.5] mm for the active (*N* = 9) versus sham (*N* = 17) group, respectively. There was a between-group difference in the change in total abdominal expiratory muscle thickness on day 3; mean (95% confidence interval (CI)) changes from baseline on day 3 were 1.76 (0.21, 3.30) versus − 0.50 (− 1.56, 0.57) mm for the active (*N* = 7) and sham (*N* = 12) group, respectively, with a treatment difference (95% CI) of 2.25 (0.34, 4.16) mm (*P* = 0.02) (Fig. 3). No between-group difference in total abdominal expiratory muscle thickness was found on day 5. Ultrasound results for all individual respiratory muscles are presented in Additional file 1: Tables 1–6; no changes between groups or over days were found.

#### Inflammatory markers

Figure 2b presents the results for plasma cytokine levels at baseline and on day 3 (*N* = active/sham; 19/17); no differences between groups or over days were found.

#### Clinical outcomes

**Fig. 4:**
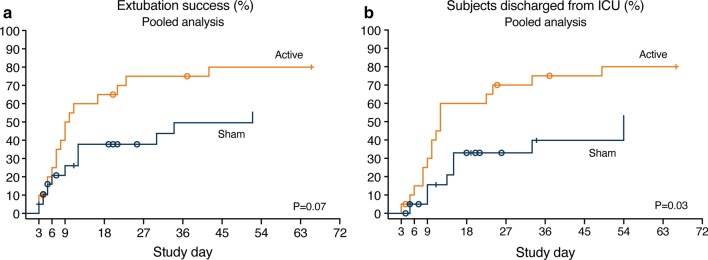
Pooled results on clinical endpoints. **a** Pooled results on extubation success. **b** Pooled results on ICU length of stay. *P* values are based on Gray’s test. Cumulative event rates were estimated based on competing risk analysis, with the competing risks of death or withdrawal of ICU treatment (e.g., ventilator support) with the intention of subsequent death. Symbols: o for competing events; + for censored data

Figure 4 shows results for extubation success and ICU length of stay. Using the cumulative incidence competing risks method for the total study population, median ventilation duration and ICU length of stay were 10 versus 52 days (*P* = 0.07), and 12 versus 54 days (*P* = 0.03) for the active versus sham group, respectively. For patients that were successfully extubated during ICU stay, the median ventilation duration was 8.5 [5.6–12.2] versus 10.5 [5.3–25.6] days (*P* = 0.60) for the active (*N* = 16) versus sham (*N* = 10) group, respectively. Median ICU length of stay was 10.5 [8.0–14.5] versus 14.0 [9.0–19.5] days (*P* = 0.36) for those active (*N* = 16) versus sham (*N* = 8) patients that were extubated and discharged alive from the ICU. During the ICU stay, 3/20 (15%) patients died in the active group, versus 8/20 (40%) patients in the sham group (*P* = 0.16).

## Discussion

The current feasibility study demonstrates that breath-synchronized expiratory muscle FES is feasible, safe and effective in eliciting expiratory muscle activity during mechanical ventilation in ICU patients. This supports the findings of the Australian study [17], but in a more heterogeneous ICU population. The pooled analysis of the Australian and Holland studies also provides important insights for the design of future studies to evaluate whether this approach could improve weaning and ventilator liberation outcome.

### Rationale for expiratory muscle FES

Recent studies suggest that maintaining diaphragm activity during mechanical ventilation minimizes diaphragm disuse atrophy and may improve clinical outcome [4, 20]. The current study was designed to investigate the feasibility and efficacy of eliciting expiratory muscle activity in the early stages of mechanical ventilation, based on the assumption that mechanical ventilation is associated with expiratory muscle disuse atrophy. While this assumption has not been extensively studied, it is known that controlled mechanical ventilation may (partly) silence respiratory centers in the brainstem, resulting in disuse of the inspiratory and expiratory muscles [21]. In addition, data on rectus abdominis biopsies show that critically ill patients exhibit smaller myofiber cross-sectional area compared with controls [22]. The effects of critical illness and mechanical ventilation on the change in expiratory muscle thickness, however, are yet unknown but may be of clinical relevance as increasing evidence demonstrates expiratory muscle weakness at the time of ventilator weaning [1, 9, 10, 13, 23], likely as a consequence of muscle disuse. Potential explanations for how expiratory muscle weakness affect weaning or extubation outcome include inadequate secretion clearance and insufficient cough capacity, resulting in respiratory complications such as pneumonia and atelectasis. Also, the expiratory muscles support inspiration in the presence of diaphragm dysfunction [1]; weakness may thus result in reduced ventilatory capacity. In line with our earlier results [17], we report more successful extubations for the pooled active group; however, this needs confirmation in an appropriately powered study.

### Feasibility, contractile response and safety

We demonstrate that expiratory muscle FES as applied twice daily in the Holland study with a maximum intensity of 100 mA is feasible and safe in selected critically ill patients and allows high therapy compliance after an adequate contractile response to FES was verified. No FES-related serious harm or complications were reported, and only few sessions were stopped early after meeting safety criteria.

Over the last decade, inconclusive evidence for the clinical benefits of FES in ICU patients was published [15, 24], mainly targeting limb muscles. One reason for these inconsistent results may be the lack of reporting of treatment compliance and contractile response to stimulation [25]. The effectiveness of FES in activating muscles at an adequate level depends on patient characteristics; factors such as sepsis, edema and vasopressor may play a role [26, 27]. No potential explanatory factors hampering contractile response were found in our patients that did not pass the expiratory muscle FES eligibility test (*N* = 2 for maximum intensity of 60 mA (no data available on whether these patients would respond at higher intensities), *N* = 3 for maximum intensity of 100 mA). Besides changes in Richmond Agitation-Sedation Scale, no clinical or ventilator parameters were associated with changes in applied stimulation intensity for the active group. The latter could be explained by the fact that stimulation intensity remained relatively stable, while ICU patients show more day-to-day variation in clinical signs.

Interestingly, our high success rate of contractile activation is not in line with Grunow et al. [25], studying contractile response to FES (verified visually or on palpation) in eight limb muscle groups and reporting that only 64.4% of applied stimulations led to an adequate response on the day of ICU admission; this number declined to 25% after one week. Although the expiratory muscles were not targeted in their study, their maximum stimulation intensity was 70 mA, i.e., our median threshold intensity. While a strong muscle contraction depends on many factors (e.g., pulse duration, skin/electrode interface, electrode size and location to motor point), it is plausible that increasing intensity would provide higher contractile response rates in their study. In addition, we used ultrasound to verify the initial response, which we consider a more objective measure compared to palpation, especially in patients with high body mass index or edema. Lastly, we did not experience decreases in contractile response throughout the study, likely due to the higher intensities used and pre-randomization stimulation titration.

Plasma cytokine levels were measured to evaluate whether early application of FES was associated with enhanced systemic inflammatory response. Starting expiratory muscle FES as early as possible is important, as respiratory muscle atrophy largely develops within the first 4 days of mechanical ventilation [20, 29]. However, it is important to assess whether patients tolerate the increased physical demands and whether early FES causes an inflammatory state. We did not find associations with systemic inflammatory response, but with the large variability in pro-inflammatory state of ICU patients, no clear implications can be generated about potential protective effects. This is in line with recent work [30] on limb muscle FES in ICU patients and with data in healthy subjects demonstrating that the effects of FES are similar to those of mild exercise [31], i.e., inhibiting pro-inflammatory cytokines [32]. Furthermore, Hickmann et al. [33] showed that early exercise during the onset of septic shock did not enhance inflammation and preserved muscle mass. Similar mechanisms may explain potential protective effects of FES on muscle loss, but this requires repeated measurements of cytokines during the full period of a FES protocol, which was not the focus of our study and would be of interest to address in future research.

There are yet no clinical applications of FES targeting the respiratory muscles of ICU patients under mechanical ventilation. Transvenous phrenic nerve pacing is currently being studied as potential intervention for improving diaphragm strength in difficult-to-wean patients (NCT03096639). However, this technique is invasive, increasing risks associated with subclavian vein cannulation and blood stream infections. In contrast, we focused on employing breath-synchronized expiratory muscle FES in the early phase of critical illness, aiming to prevent (or attenuate) the development of muscle disuse in selected patients with an anticipated expected prolonged duration of mechanical ventilation. We reason that this could be a novel noninvasive application within a respiratory muscle-protective ventilation strategy.

### Potential effects on clinical endpoints

For the pooled data, we observed a between-group difference in total abdominal expiratory muscle thickness changes on day 3, favoring the active group. Although effects were small, our observations are in line with Dall’Acqua et al. [28], showing that FES of the rectus abdominis muscle (note that this muscle is to a limited extent involved in expiration [1], therefore not specifically targeted in our study) of ICU patients resulted in muscle mass preservation in the active group, while thickness decreased in the sham group. However, we found no between-group differences on day 5, likely resulting from insufficient sample size and observer variability (see ‘Strengths and limitations’ section).

In the pooled analysis of clinical outcomes using the cumulative incidence competing risks method, we report between-group differences in median ventilation duration and ICU length of stay. As the incidence rate of the event of interest was influenced by competing events (particularly death in the sham group) and the estimates might not be stable given the small sample size, we also calculated the median ventilation duration and ICU length of stay for those patients that experienced the event of interest. No between-group differences were found for these subgroups. A next step would be to test the treatment effect of expiratory muscle FES in a study powered on clinical endpoints. Assuming a median effect size with 60% of patients successfully extubated on day 9 for the intervention group versus 45% of patients in the sham group, a next study would require 254 participants (hazard approach; two-sided log-rank test with two-sided alpha of 0.05, beta of 0.1, mortality on day 9 of 20%, and 10% of patients lost to follow-up).

### Strengths and limitations

Strengths are that we enrolled a heterogeneous group of patients from different centers and performed a relatively high number (*n* = 272) of FES sessions. Also, contractile response was verified prior to randomization, resulting in high treatment compliance and to ensure that neuromuscular status was comparable between groups. Lastly, a pooled analysis was performed, including evaluation of cytokines, to assess potential benefits in a larger cohort.

This study has some limitations. First, it was designed with the assumption that ultrasound could provide sufficient insights into the effects of expiratory muscle FES on muscle mass preservation. Despite using a standardized protocol [19], obtaining reliable ultrasound measurements in ICU patients can be challenging. Changes in expiratory muscle thickness can reflect actual changes in muscle thickness (i.e., atrophy or hypertrophy), but could also be affected by observer variability and patient characteristics. For example, motion of abdominal contents with respiration could passively stretch the abdominal expiratory muscles. Also, abdominal expiratory muscles have more degrees of freedom to move compared to the diaphragm; active contraction of one muscle layer could directly influence the position of the adjacent layer. For this reason, we evaluated changes in total abdominal expiratory muscle thickness and used a linear mixed model to account for individual changes, but the study was not powered sufficiently to draw any conclusions on these results. In contrast, ultrasound is valuable for verifying contractile response to stimulation (Additional file 1: Figure 4). Second, sample size was small and patients were enrolled relatively late after intubation. This resulted in a highly selected study population prone to prolonged mechanical ventilation. Because of this reason, the absence of protocolized ventilator and weaning strategies, and post-randomization events (particularly death in the sham group), results on clinical outcomes should be interpreted with caution and generalizability of the findings is limited. Third, this study lacks a robust outcome parameter to assess physiological effects of expiratory muscle FES, including dose–response relationships. The dosage of 30-min stimulation sessions twice daily was chosen based on a few practical and physiological considerations. First, while the rate of expiratory muscle atrophy is yet unknown, diaphragm atrophy rapidly occurs after the start of mechanical ventilation [20, 29]. Hence, we wanted to limit the delay between stimulation sessions to a maximum duration of 24 h, which is only possible to guarantee by including more than one stimulation session per day. Second, it is known that the force evoked from a muscle by electrical stimulation could decline rapidly over time because of repetitive activation of the same motor units. We therefore reasoned that limiting the stimulation session duration to 30 min would help to ensure a strong muscle contraction throughout the session. Other considerations included availability of study personnel and possible interference with clinical protocols/activities. Nevertheless, a next study should consider different FES protocols in order to find the optimal session frequency and duration for improving clinical outcomes. Moreover, although it would be interesting to assess muscle changes on a cellular or functional level and in response to different expiratory muscle FES protocols, such measurements would require repeated muscle biopsies or assessment of gastric twitch pressures in response to stimulation of the expiratory muscle nerve roots, respectively. We did not perform these invasive and technically challenging techniques in our study, but focused on the feasibility and efficacy of employing expiratory muscle FES in the early phase of ICU stay. Addressing such physiological endpoints would be of interest in order to better understand the potential effects of expiratory muscle FES on maintaining muscle function.

## Conclusion

Breath-synchronized expiratory muscle FES is a feasible and generally safe intervention to elicit expiratory muscle activity during the early stages of mechanical ventilation in selected ICU patients. This could be a novel intervention within a respiratory muscle-protective ventilation strategy. The effects of expiratory muscle FES on weaning and ventilator liberation outcome remain to be studied.

## Supplementary information

**Additional file 1.** Extended methods and results.

## Data Availability

The datasets and protocols used and analyzed during the current study are available from the corresponding author on reasonable request.

## Abbreviations

FES: Functional electrical stimulation
ICU: Intensive care unit
IL: Interleukin
PSV: Pressure support ventilation
TNF: Tumor necrosis factor
