# Supplementary material for: Breath-synchronized electrical stimulation of the expiratory muscles in mechanically ventilated patients: a randomized controlled feasibility study and pooled analysis
Source: Crit Care. 2020 Oct 30;24:628. doi: 10.1186/s13054-020-03352-0 (PMC7596623; doi:10.1186/s13054-020-03352-0)
Supplement: Supplementary file 1 — Additional file 1. Extended methods and results. [file 13054_2020_3352_MOESM1_ESM.docx]

**Breath-synchronized electrical stimulation of the expiratory muscles in mechanically ventilated patients: a randomized controlled feasibility study and pooled analysis**

ADDITIONAL FILE: EXTENDED METHODS AND RESULTS

Annemijn H. Jonkman, MSc.^1,2^; Tim Frenzel, MD PhD^3^; Euan J. McCaughey, PhD^4,5^; Angus J. McLachlan, PhD^6^; Claire L. Boswell-Ruys, PhD^4,5^; David W. Collins, MD^7^; Simon C. Gandevia, MD PhD^4,5^; Armand R.J. Girbes, MD PhD^1,2^; Oscar Hoiting, MD^8^; Matthijs Kox, PhD^3^; Eline Oppersma, PhD^9^; Marco Peters, MD^8^; Peter Pickkers, MD PhD^3^; Lisanne H. Roesthuis, MSc^3^; Jeroen Schouten, MD PhD^3^; Zhong-Hua Shi, MD^1,2^; Peter H. Veltink, PhD^10^; Heder J. de Vries, MD^1,2^; Cyndi Shannon Weickert, PhD^4,11,12^; Carsten Wiedenbach, MSc^8^; Yingrui Zhang, MD^1^; Pieter R Tuinman, MD PhD^1,2^; Angélique M.E. de Man, MD PhD^1,2^; Jane E. Butler, PhD^4,5^; and Leo M.A. Heunks, MD PhD^1,2^

**Affiliations**

1: Department of Intensive Care Medicine, Amsterdam University Medical Centers, location VUmc, Amsterdam, The Netherlands

2: Amsterdam Cardiovascular Sciences Research Institute, Amsterdam UMC, Amsterdam, the Netherlands

3: Department of Intensive Care Medicine, Radboud University Medical Center, Nijmegen, The Netherlands

4: Neuroscience Research Australia, 139 Barker Street, Randwick, NSW 2031, Australia.

5: School of Medical Sciences, University of New South Wales, Kensington, NSW 2052, Australia.

6: Liberate Medical LLC, Crestwood, KY 40014, USA

7: Prince of Wales Hospital, Randwick, NSW, 2031, Australia

8: Department of Intensive Care Medicine, Canisius Wilhelmina Hospital, Nijmegen, The Netherlands

9: Cardiovascular and Respiratory Physiology group, Technical Medical Centre, University of Twente, Enschede, The Netherlands

10: Department of Biomedical Signals & Systems, Technical Medical Centre, University of Twente, Enschede, The Netherlands.

11: School of Psychiatry, University of New South Wales, Kensington, NSW 2052, Australia.

12: Department of Neuroscience and Physiology, Upstate Medical University, New York 13210, USA

**Corresponding author:** Prof. Leo Heunks, MD, PhD, E-mail: [L.Heunks@amsterdamumc.nl](mailto:L.Heunks@amsterdamumc.nl). Amsterdam UMC, location VUmc, Postbox 7505, 1007 MB Amsterdam, The Netherlands.

**EXTENDED METHODS**

**Holland study**

*Investigational device*

The investigational product used in this study was the VentFree prototype (VF03-K), an electrotherapy device developed by Liberate Medical LLC, USA. VentFree is an electrotherapy device that:

1) monitors the user’s voluntary breathing activity using an airflow sensor (which will be placed in line with the expiratory limb of the ventilator), and

2) applies FES to the expiratory muscles over two stimulation channels, during the expiratory phase of breathing.

The VF03-K prototype consisted of a stimulation trigger system developed by Liberate Medical, a CE-marked variable orifice pneumotachograph (Model 3100, Braebon Medical), CE-marked transcutaneous stimulation electrodes (Ultrastim, Axelgaard Manufacturing), and an FDA-cleared neuromuscular electrical stimulator (Continuum, Empi). An overview of the prototype is shown in **e-Figure 1**.

The pneumotachograph was fitted in line with the expiratory limb of the subject’s mechanical ventilator. Stimulation was automatically applied during exhalation, based on the measured flow. As per the manufacturer’s instructions, the pneumotachograph component was for single patient use. In addition, two hydrophobic filters were placed between the pneumotachograph pressure ports and the tubing that connects the pneumotachograph to the stimulation trigger. Flow resistance was approximately 1.0 mbar per 60 lpm.

*Placement of electrodes*

A new set of electrodes was used for each subject enrolled in the study. To optimize electrical conduction, the skin was shaved (if needed) and rinsed with soap and water before applying the electrodes on dry skin. Electrodes were positioned at the postolateral side of the abdominal wall, such that the transverse abdominal (TrA), internal oblique (IO), and external oblique (EO) muscles were be stimulated. The rectus abdominis (RA) muscle was not directly stimulated since this muscle mainly provides stability to the abdominal wall and is less involved in forced exhalation as compared to the TrA, IO, and EO muscles. In general, the positioning of the anodes was directed form the midline, 2 cm below and parallel to the costal margin, angled obliquely toward the anterior superior iliac spine. The position of the cathodes was directed from the midaxillary line at the level of the 8th thoracic vertebral body, angled directly toward the posterior superior iliac spine (see **e-Figure 2a**). The electrodes were positioned symmetrically on both sides of the abdomen. This configuration has been used in a study of Lim et al.(1) and McCaughey and colleagues (2), where the use of FES of expiratory muscles to assist respiration was investigated. Small adaptations of the electrode configuration were possible based on individual anatomical differences. In addition, if it was not possible to turn the patient, an alternative electrode position was used (**e-Figure 2b**).

The position of the electrodes was re-marked daily with a semi-permanent marker to maintain the same location of stimulation for each session. Proper electrode adherence to the skin was assessed prior to each stimulation session and electrodes were removed and replaced if needed (e.g., if the adhesive gel became dry or if the electrodes did not properly stick to the skin). At minimum the electrodes were replaced every five stimulation sessions in order to minimize the risk of skin burns caused by electrical stimulation.

*Confirmation of eligibility to expiratory muscle FES*

Prior to randomization, a first expiratory muscle FES test was performed to confirm eligibility for the intervention. Trains of biphasic rectangular stimuli (frequency; 30 Hz, pulse width 352 µs, same as the FES settings used for the active group) with incremental intensities (starting at 10 mA, steps of 5 mA) were applied to determine the threshold current for tetanic muscle contraction and the maximum tolerated intensity. The maximum intensity was initially set to 60 mA and upon enrolment increased to 100 mA (see Manuscript – *Methods*). Stimulation increments were made based on clinical judgment of comfort (based on judgment of the nurse and/or present physician, or a RASS score of ≥2). If FES resulted in patient discomfort, stimulation intensity was not further increased. If the threshold intensity for contraction was not reached at this point, the subject was withdrawn from the study. Patients were also withdrawn from the study if stimulations with maximum intensity did not result in any visible contraction of the expiratory muscles. Subjects were replaced until twenty patients were successfully randomized and received at least one 30-min stimulation session.

Contraction of the expiratory muscles in response to FES was verified with ultrasound. A linear ultrasound probe was placed on the abdominal wall parallel to the stimulation electrodes in order to visualize the EO, IO and TrA muscles. The response to stimulation was evaluated for both the left- and right-sided expiratory muscles. An example of a visible contraction in response to stimulation is presented in **e-Figure 5**.

*Stop criteria for expiratory muscle FES*

Sessions were stopped early in the presence of at least one of the following changes in vital signs: heart rate < 40 bpm or > 130 bpm; mean arterial pressure < 60 mmHg or > 110 mmHg, respiratory rate > 40 breaths/min, SpO2 < 90%, behavior pain scale > 4 or patient request to stop.

*Randomization and blinding*

Patients were randomized to receive active (intervention) of sham (control) expiratory muscle FES, using an online randomization tool, with a 1:1 allocation ratio using blocks of four patients. Randomization was performed by the researcher administering expiratory muscle FES. Patients, caregivers and outcome assessors were blinded to the treatment allocation. The researcher administering expiratory muscle FES did not perform ultrasound recordings. Outcome assessors were not in the patient room when stimulation was delivered.

*Outcome measurements*

***Ultrasound.*** Muscle thickness of the expiratory muscles and diaphragm was determined on B-mode ultrasound images, obtained with a linear high-frequency (>7.5 MHz) transducer. Ultrasound was performed at baseline (before the first FES session), in between FES sessions at every Monday, Wednesday and Friday, within 24 hours after extubation, and at ICU discharge. Although both sides were electrically activated, only right-sided muscles were evaluated with ultrasound, assuming anatomical symmetry with left-sided muscles. A standardized ultrasound protocol was used.(3) For all measurements, the probe was placed perpendicular to the skin. To visualize the RA muscle, the transducer was positioned in a transverse orientation approximately 2-3 cm above the umbilicus, 2-3 cm lateral from the midline. Maximum thickness of the RA muscle was subsequently obtained by displacing the probe in cranial-caudal directions. When moving further laterally, the EO, IO and TrA muscles were identified as three parallel hypoechogenic layers, enclosed by clear bright lines representing muscle fascia sheaths. This was approximately at the anterior axillary line, midway between the inferior border of the rib cage and the iliac crest. Minimal pressure was applied to the skin to prevent muscle deformation. The diaphragm was visualized in the zone of apposition, with the probe placed on the anterior axillary line, between the 8^th^ and 11^th^ rib. For all measurements, a video of at least three breath cycles was captured and stored for offline analysis. From the ultrasound videos, thickness of the RA, EO, IO, and TrA muscles was measured as the distance between the inner edges of the superior and inferior fascia sheets, perpendicular to the muscle direction. Total lateral expiratory muscle thickness was measured as the distance between the upper EO fascia and lower TrA fascia (see **e-Figure 5**). Diaphragm thickness was obtained as the distance between the diaphragmatic pleura and peritoneum (for reference to these methods, see (3)). For each muscle, the average thickness value of three consecutive breaths was calculated for both the end-inspiratory and end-expiratory phase of breathing. For the expiratory muscles, data on end-inspiratory muscle thickness (resting position) were used to assess changes in thickness over time. For assessment of diaphragm thickness changes over time, data on end-expiratory thickness were used.

***Cytokines.*** At baseline and on the third treatment day (within 72h after the first FES session), peripheral blood samples were drawn into EDTA tubes and centrifuged immediately (for 10 minutes, at 21 degrees of Celsius and with 1800G). The plasma was frozen in aliquot tubes and stored at -80 degrees of Celsius until pooled analysis (see below).

*Data analysis and statistics*

Statistical analysis followed similar methods as described in our previous work (4). Categorical data are summarized in terms of the number of participants with data at the relevant time point (n) and as a percentage of all participants. Continuous data are expressed as median [25^th^ - 75^th^ percentiles] or mean and standard deviation, unless otherwise specified. Statistical analyses were performed using two-sided hypothesis tests at the overall 5% significance level. SAS (SAS software, version 9.3) and SPSS (SPSS IBM Statistics, version 26) were used to perform all summaries and analyses. Results were visualized using GraphPad Prism (Prism, version 8.2.1).

As this is a feasibility study and no data were available on the effects of expiratory muscle FES on expiratory muscle thickness when designing the study, we planned to enroll a convenience sample of ten subjects per group, twenty in total.

***Feasibility.*** Compliance to the expiratory muscle FES session was calculated as the percentage of all sessions that should have been completed between randomization and completion or withdrawal and was considered a continuous variable.

For the active group, changes in applied stimulation intensity over the study period were calculated per subject as the percentage change in intensity between baseline and the subject’s last expiratory muscle FES session. Session-to-session changes in stimulation intensity were calculated as the geometric mean of the ratio of change, in which the ratio of change between two consecutive sessions was assessed relative to the lowest intensity that was applied. Thus, a 5% change in intensity between session X and the previous session X-1 could mean that at session X, the stimulation intensity was either 5% higher or lower compared to session X-1. Associations between RASS and applied stimulation intensity (**e-Figure 4**) were analyzed with a mixed model design, using a random effect of participant and fixed effect of RASS.

***Cytokines.*** Changes in plasma cytokine levels were assessed using a two-way ANOVA with factors of group (active, sham) and time (measurements at baseline and on the third treatment day). Measured cytokines included tumor necrosis factor (TNF)-α, interleukin (IL)-1 receptor antagonist, IL-1β, IL-6, IL-8 and IL-10. Subjects with a missing blood sample at one of the time points were excluded from the analysis. For those patients with a plasma cytokine level below the limit of detection, the value corresponding to this limit of detection was used for further analyses. A log transformation was applied as data did not fit a normal distribution. In the presence of an interaction effect with P<0.05, within-group differences were evaluated with a paired t-test.

**Pooled analysis**

*Cytokines*

After the last subject was enrolled, the frozen blood samples of the Australian study were shipped on dry ice to The Netherlands, such that cytokine levels were analyzed in one batch, using a multiplex Luminex assay, according to the manufacterer’s instruction (Millipore, Billerica, USA).

*Statistics*

***Ultrasound data.*** Change from baseline of the total expiratory muscles, internal oblique, external oblique, transversus abdominis, rectus abdominis, and diaphragm thickness were treated as continuous variables and analyzed using a linear mixed effects model with fixed factors of baseline thickness, treatment, assessment session and treatment by assessment session interaction, and a random effect of participant (change from baseline thickness ~ baseline thickness + treatment + assessment session + (treatment * assessment session) + (1|patient)). These mixed models compare the thickness of the muscles over time. Due to the small sample size, and the risk of a normality test being underpowered, we followed the statistical methods in Dall’ Acqua et al.(5) where the distribution of muscle thickness was assumed normal.

***Clinical endpoints.*** Ventilation duration and ICU length-of-stay were analyzed using Gray’s test in the survival analysis, with the competing risks of death or withdrawal of treatment (e.g. ventilator support) with the intention of subsequent death. Gray’s test compares cause-specific cumulative incidence curves. Median ventilation duration (days) was defined as the timepoint when 50% of the participants experienced the event of interest. In the case where less than 50% of participants achieved the outcome, due to either competing events or censoring, the median time to the outcome was not estimable. In addition, we calculated the median [q_1_-q_3_] ventilation duration and ICU length-of-stay for those patients that experienced the event of interest during the study period. Differences between these groups were assessed with the Mann-Whitney U test, according to the distribution.

***Cytokines.*** Analysis of plasma cytokine levels followed the same approach as described for the Holland study.

**FIGURE LEGENDS**

**E-figure 1.** Picture of the VentFree prototype device, model VF03-K (labeled as Triggering Device). This device is connected to the flow sensor that was placed in line with the ventilator tubing. The red connection cable connects the triggering device to the stimulator (a commercially available Empi Continuum stimulator was used for this study). When expiratory flow is detected by the triggering device, it automatically triggers the stimulator to apply FES to the expiratory muscles via surface electrodes placed over the abdominal wall.

**E-figure 2. a.** Primary position for electrode placement. “A” electrodes were placed starting at the anterior superior illiac spine and moving up toward the xiphoid process. “B” electrodes were positioned starting at the level of the eighth thoracic vertebra (T8) on the mid axillary line and extending down toward posterior superior iliac spine. **b.** Alternative electrode position that was used when it was not possible to place the electrodes in the primary position (e.g. when it was not possible to turn the patient). The top half of the top electrode overlapped slightly with the bottom of the rib cage. The bottom electrode was placed between the bottom of the top electrode and the top of the hip bone.

**E-figure 3**. Relative stimulation intensity categorized by Richmond Agitation-Sedation Scale (RASS). 100% corresponds to the patient’s average intensity that was applied during the study period. Data are presented as mixed model estimated means with 95% confidence interval (P=0.02 for the fixed effect of RASS).

**E-figure 4.** Example of a representative patient that responded to expiratory muscle FES. Stimulation intensity for this example was 50 mA, resulting in a FES-induced increase in total expiratory muscle thickness from 10.1 mm to 11.3 mm. EO: external oblique muscle, IO: internal oblique muscle, TrA: transversus abdominis muscle. Total expiratory muscle thickness was measured as the distance between the upper EO fascia and lower TrA fascia.


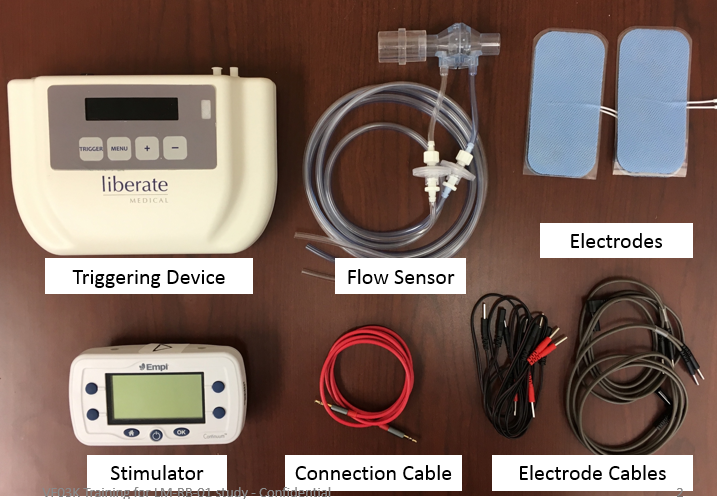


**E-figure 1.** Picture of the VentFree prototype device, model VF03-K (labeled as Triggering Device). This device is connected to the flow sensor that was placed in line with the ventilator tubing. The red connection cable connects the triggering device to the stimulator (a commercially available Empi Continuum stimulator was used for this study). When expiratory flow is detected by the triggering device, it automatically triggers the stimulator to apply FES to the expiratory muscles via surface electrodes placed over the abdominal wall.


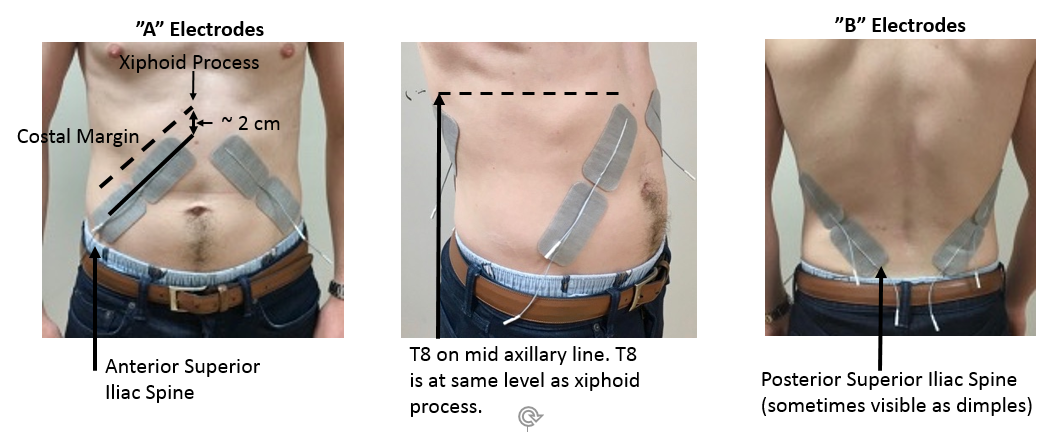


**a.**


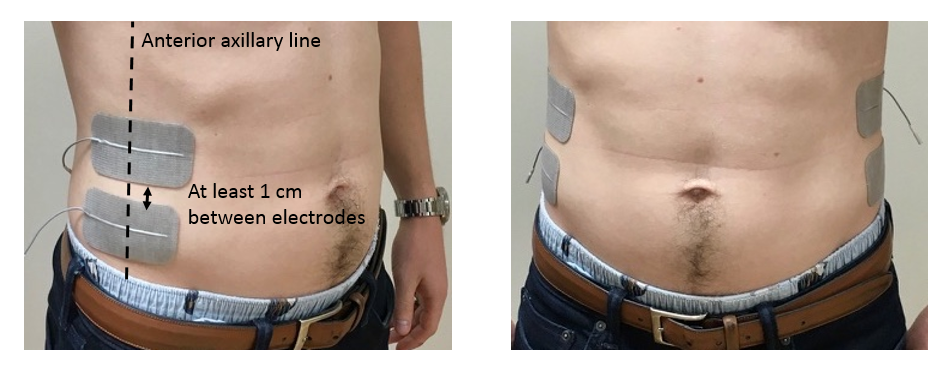


**b.**

**E-figure 2. a.** Primary position for electrode placement. “A” electrodes were placed starting at the anterior superior illiac spine and moving up toward the xiphoid process. “B” electrodes were positioned starting at the level of the eighth thoracic vertebra (T8) on the mid axillary line and extending down toward posterior superior iliac spine. **b.** Alternative electrode position that was used when it was not possible to place the electrodes in the primary position (e.g. when it was not possible to turn the patient). The top half of the top electrode overlapped slightly with the bottom of the rib cage. The bottom electrode was placed between the bottom of the top electrode and the top of the hip bone.


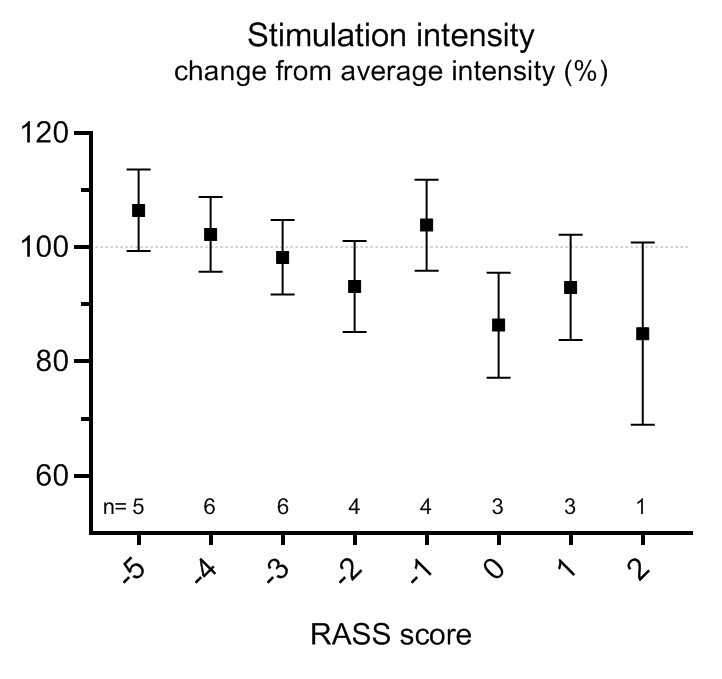


**E-figure 3**. Relative stimulation intensity categorized by Richmond Agitation-Sedation Scale (RASS). 100% corresponds to the patient’s average intensity that was applied during the study period. Data are presented as mixed model estimated means with 95% confidence interval (P=0.02 for the fixed effect of RASS).


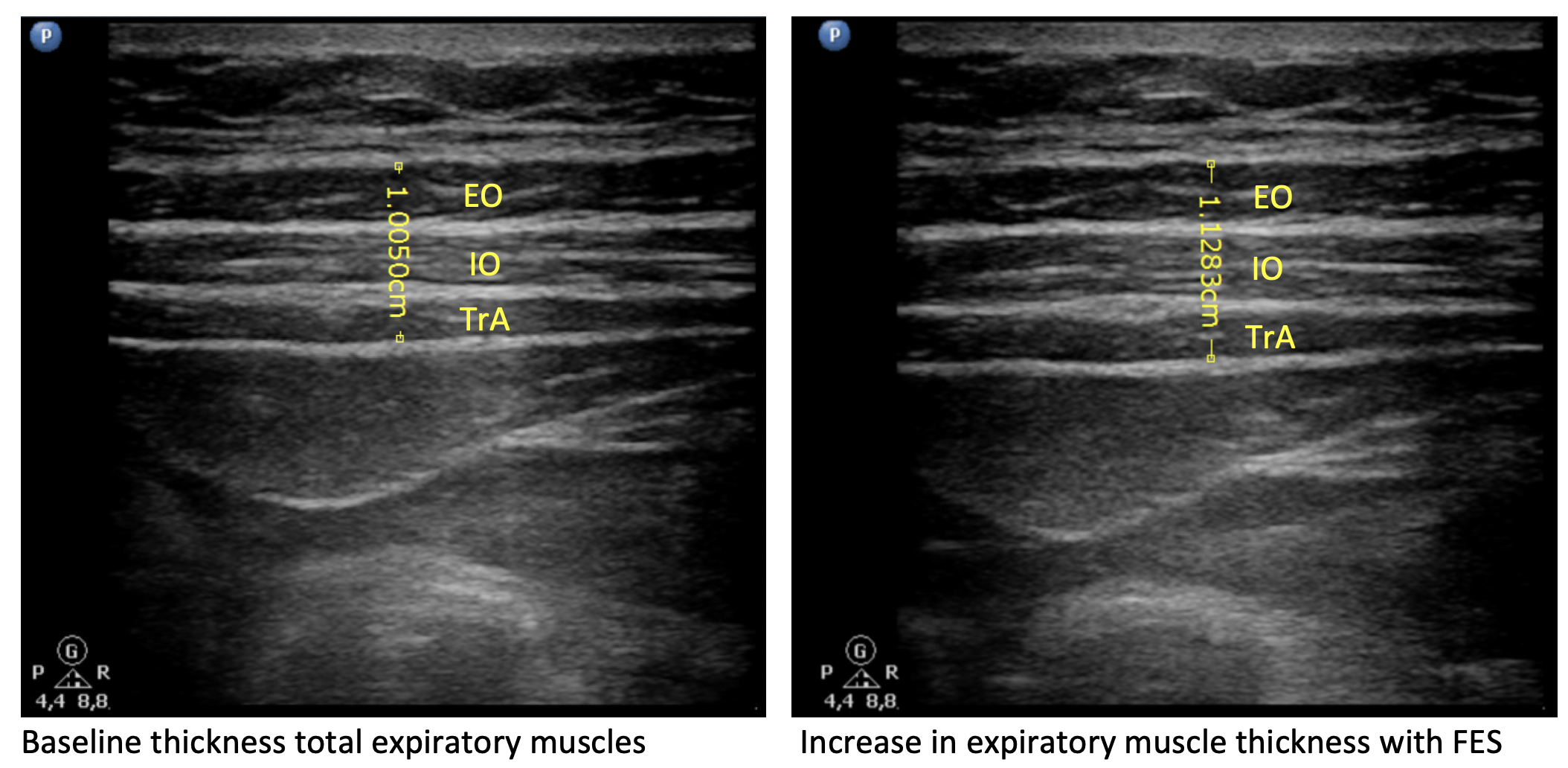


**E-figure 4**. Example of data from a representative patient that responded to expiratory muscle FES. Stimulation intensity for this example was 50 mA, resulting in a FES-induced increase in total expiratory muscle thickness from 10.1 mm to 11.3 mm. EO: external oblique muscle, IO: internal oblique muscle, TrA: transversus abdominis muscle. Total expiratory muscle thickness was measured as the distance between the upper EO fascia and lower TrA fascia.

**TABLE LEGENDS**

**E-table 1.** Pooled analysis: Mean change in end-inspiratory total expiratory muscle thickness (in mm) over the first five treatment days.

**E-table 2.** Pooled analysis: Mean change in end-inspiratory external oblique muscle thickness (in mm) over the first five treatment days.

**E-table 3.** Pooled analysis: Mean change in end-inspiratory internal oblique muscle thickness (in mm) over the first five treatment days.

**E-table 4.** Pooled analysis: Mean change in end-inspiratory transversus abdominis muscle thickness (in mm) over the first five treatment days.

**E-table 5.** Pooled analysis: Mean change in end-inspiratory rectus abdominis muscle thickness (in mm) over the first five treatment days.

**E-table 6.** Pooled analysis: Mean change in end-expiratory diaphragm thickness (in mm) over the first five treatment days.

| **E-table 1.** Pooled analysis: Mean change in end-inspiratory total expiratory muscle thickness (in mm) over the first five treatment days. | | |
| --- | --- | --- |
|  | **Sham (N=17)** | **Active (N=16)** |
| P-value for Treatment by Visit Interaction: 0.9625 |  | |
|  |  |  |
| Day 3 Change from Baseline (n) | 12 | 6 |
| Mean (95% CI) | -0.39 (-1.58, 0.79) | 1.56 (-0.71, 3.83) |
| LS Mean (95% CI) | -0.50 (-1.56, 0.57) | 1.76 (0.21, 3.30) |
| Treatment Difference (95% CI) | 2.25 (0.34, 4.16) | |
| P-value | 0.0234 | |
| Day 5 Change from Baseline (n) | 11 | 6 |
| Mean (95% CI) | -0.35 (-1.52, 0.82) | 0.14 (-1.34, 1.62) |
| LS Mean (95% CI) | -0.51 (-1.63, 0.60) | 0.34 (-1.21, 1.89) |
| Treatment Difference (95% CI) | 0.85 (-1.09, 2.80) | |
| P-value | 0.3690 | |
| All statistics except for Mean (95% CI) are resulted from a linear mixed model, with the fixed factors of baseline thickness treatment, visit and treatment by visit interaction, and a random effect of participant. | | |

| **E-table 2.** Pooled analysis: Mean change in end-inspiratory external oblique muscle thickness (in mm) over the first five treatment days. | | |
| --- | --- | --- |
|  | **Sham (N=17)** | **Active (N=16)** |
| P-value for Treatment by Visit Interaction: 0.5955 |  | |
|  |  |  |
| Day 3 Change from Baseline (n) | 12 | 6 |
| Mean (95% CI) | 0.09 (-0.40, 0.59) | 0.32 (-0.44, 1.08) |
| LS Mean (95% CI) | -0.01 (-0.43, 0.41) | 0.58 (-0.04, 1.20) |
| Treatment Difference (95% CI) | 0.59 (-0.18, 1.36) | |
| P-value | 0.1235 | |
| Day 5 Change from Baseline (n) | 11 | 6 |
| Mean (95% CI) | -0.25 (-0.79, 0.28) | 0.19 (-0.55, 0.94) |
| LS Mean (95% CI) | -0.37 (-0.81, 0.08) | 0.46 (-0.16, 1.08) |
| Treatment Difference (95% CI) | 0.82 (0.04, 1.60) | |
| P-value | 0.0407 | |
| All statistics except for Mean (95% CI) are resulted from a linear mixed model, with the fixed factors of baseline thickness treatment, visit and treatment by visit interaction, and a random effect of participant. | | |

| **E-table 3.** Pooled analysis: Mean change in end-inspiratory internal oblique muscle thickness (in mm) over the first five treatment days. | | |
| --- | --- | --- |
|  | **Sham (N=17)** | **Active (N=16)** |
| P-value for Treatment by Visit Interaction: 0.3840 |  | |
|  |  |  |
| Day 3 Change from Baseline (n) | 12 | 6 |
| Mean (95% CI) | 0.01 (-0.64, 0.65) | 0.37 (-1.07, 1.82) |
| LS Mean (95% CI) | -0.06 (-0.83, 0.71) | 0.50 (-0.63, 1.62) |
| Treatment Difference (95% CI) | 0.56 (-0.83, 1.94) | |
| P-value | 0.4074 | |
| Day 5 Change from Baseline (n) | 11 | 6 |
| Mean (95% CI) | 0.16 (-0.78, 1.11) | -0.40 (-1.57, 0.77) |
| LS Mean (95% CI) | 0.03 (-0.77, 0.84) | -0.28 (-1.40, 0.85) |
| Treatment Difference (95% CI) | -0.31 (-1.72, 1.09) | |
| P-value | 0.6459 | |
| All statistics except for Mean (95% CI) are resulted from a linear mixed model, with the fixed factors of baseline thickness treatment, visit and treatment by visit interaction, and a random effect of participant. | | |

| **E-table 4.** Pooled analysis: Mean change in end-inspiratory transversus abdominis muscle thickness (in mm) over the first five treatment days. | | |
| --- | --- | --- |
|  | **Sham (N=17)** | **Active (N=16)** |
| P-value for Treatment by Visit Interaction: 0.8495 |  | |
|  |  |  |
| Day 3 Change from Baseline (n) | 12 | 6 |
| Mean (95% CI) | -0.24 (-0.67, 0.20) | 0.37 (-0.16, 0.89) |
| LS Mean (95% CI) | -0.27 (-0.67, 0.14) | 0.36 (-0.23, 0.94) |
| Treatment Difference (95% CI) | 0.62 (-0.09, 1.33) | |
| P-value | 0.0828 | |
| Day 5 Change from Baseline (n) | 11 | 6 |
| Mean (95% CI) | -0.23 (-0.69, 0.24) | 0.18 (-0.72, 1.07) |
| LS Mean (95% CI) | -0.27 (-0.69, 0.15) | 0.17 (-0.42, 0.75) |
| Treatment Difference (95% CI) | 0.44 (-0.29, 1.16) | |
| P-value | 0.2240 | |
| All statistics except for Mean (95% CI) are resulted from a linear mixed model, with the fixed factors of baseline thickness treatment, visit and treatment by visit interaction, and a random effect of participant. | | |

| **E-table 5.** Pooled analysis: Mean change in end-inspiratory rectus abdominis muscle thickness (in mm) over the first five treatment days. | | |
| --- | --- | --- |
|  | **Sham (N=17)** | **Active (N=16)** |
| P-value for Treatment by Visit Interaction: 0.5412 |  | |
|  |  |  |
| Day 3 Change from Baseline (n) | 12 | 8 |
| Mean (95% CI) | -0.27 (-0.69, 0.15) | 0.05 (-0.61, 0.71) |
| LS Mean (95% CI) | -0.21 (-0.67, 0.26) | 0.04 (-0.54, 0.63) |
| Treatment Difference (95% CI) | 0.25 (-0.51, 1.01) | |
| P-value | 0.4960 | |
| Day 5 Change from Baseline (n) | 10 | 6 |
| Mean (95% CI) | -0.01 (-0.72, 0.70) | 0.01 (-0.80, 0.82) |
| LS Mean (95% CI) | 0.02 (-0.49, 0.53) | -0.08 (-0.74, 0.58) |
| Treatment Difference (95% CI) | -0.10 (-0.95, 0.75) | |
| P-value | 0.8084 | |
| All statistics except for Mean (95% CI) are resulted from a linear mixed model, with the fixed factors of baseline thickness treatment, visit and treatment by visit interaction, and a random effect of participant. | | |

| **E-table 6.** Pooled analysis: Mean change in end-expiratory diaphragm thickness (in mm) over the first five treatment days. | | |
| --- | --- | --- |
|  | **Sham (N=17)** | **Active (N=16)** |
| P-value for Treatment by Visit Interaction: 0.9933 |  | |
|  |  |  |
| Day 3 Change from Baseline (n) | 7 | 9 |
| Mean (95% CI) | -0.17 (-0.35, 0.01) | -0.15 (-0.34, 0.04) |
| LS Mean (95% CI) | -0.20 (-0.39, 0.00) | -0.16 (-0.33, 0.02) |
| Treatment Difference (95% CI) | 0.04 (-0.23, 0.31) | |
| P-value | 0.7515 | |
| Day 5 Change from Baseline (n) | 6 | 8 |
| Mean (95% CI) | -0.13 (-0.35, 0.09) | -0.04 (-0.39, 0.30) |
| LS Mean (95% CI) | -0.09 (-0.30, 0.13) | -0.03 (-0.21, 0.16) |
| Treatment Difference (95% CI) | 0.06 (-0.22, 0.34) | |
| P-value | 0.6619 | |
| All statistics except for Mean (95% CI) are resulted from a linear mixed model, with the fixed factors of baseline thickness treatment, visit and treatment by visit interaction, and a random effect of participant. | | |

**References**

1. Lim J, Gorman RB, Saboisky JP, Gandevia SC, Butler JE. Optimal electrode placement for noninvasive electrical stimulation of human abdominal muscles. J Appl Physiol (1985). 2007;102(4):1612-7.

2. McCaughey EJ, Boswell-Ruys CL, Hudson AL, Gandevia SC, Butler JE. Optimal electrode position for abdominal functional electrical stimulation. J Appl Physiol (1985). 2018;125(4):1062-8.

3. Tuinman PR, Jonkman AH, Dres M, Shi ZH, Goligher EC, Goffi A, et al. Respiratory muscle ultrasonography: methodology, basic and advanced principles and clinical applications in ICU and ED patients-a narrative review. Intensive Care Med. 2020.

4. McCaughey EJ, Jonkman AH, Boswell-Ruys CL, McBain RA, Bye EA, Hudson AL, et al. Abdominal functional electrical stimulation to assist ventilator weaning in critical illness: a double-blinded, randomised, sham-controlled pilot study. Crit Care. 2019;23(1):261.

5. Dall' Acqua AM, Sachetti A, Santos LJ, Lemos FA, Bianchi T, Naue WS, et al. Use of neuromuscular electrical stimulation to preserve the thickness of abdominal and chest muscles of critically ill patients: A randomized clinical trial. J Rehabil Med. 2017;49(1):40-8.
